# Hypoxanthine Phosphoribosyl Transferase 1 Is Upregulated, Predicts Clinical Outcome and Controls Gene Expression in Breast Cancer

**DOI:** 10.3390/cancers12061522

**Published:** 2020-06-10

**Authors:** Melina J. Sedano, Enrique I. Ramos, Ramesh Choudhari, Alana L. Harrison, Ramadevi Subramani, Rajkumar Lakshmanaswamy, Mina Zilaie, Shrikanth S. Gadad

**Affiliations:** 1 Center of Emphasis in Cancer, Department of Molecular and Translational Medicine, Paul L. Foster School of Medicine, Texas Tech University Health Sciences Center El Paso, El Paso, TX 79905, USA; melina.j.sedano@ttuhsc.edu (M.J.S.); enrique.ramos@ttuhsc.edu (E.I.R.); ramesh.choudhari@ttuhsc.edu (R.C.); Alana.L.Harrison@ttuhsc.edu (A.L.H.); Ramadevi.Subramani@ttuhsc.edu (R.S.); rajkumar.lakshmanaswamy@ttuhsc.edu (R.L.); 2 Department of Molecular and Translational Medicine, Paul L. Foster School of Medicine, Texas Tech University Health Sciences Center El Paso, El Paso, TX 79905, USA; 3 Graduate School of Biomedical Sciences, Texas Tech University Health Sciences Center El Paso, El Paso, TX 79905, USA; Mina.Zilaie@ttuhsc.edu

**Keywords:** *HPRT1*, cancer, genomics, breast cancer

## Abstract

Hypoxanthine phosphoribosyl transferase 1 (*HPRT1*) is traditionally believed to be a housekeeping gene; however, recent reports suggest that it is upregulated in several cancers and is associated with clinical outcomes. *HPRT1* is located on chromosome X and encodes the HPRT enzyme, which functions in recycling nucleotides to supply for DNA and RNA synthesis in actively dividing cells. Here, we used transcriptomic analyses to interrogate its expression across all known cancer types and elucidated its role in regulating gene expression in breast cancer. We observed elevated *HPRT1* RNA levels in malignant tissues when compared to normal controls, indicating its potential as a diagnostic and prognostic marker. Further, in breast cancer, the subtype-specific analysis showed that its expression was highest in basal and triple-negative breast cancer, and *HPRT1* knockdown in breast cancer cells suggested that *HPRT1* positively regulates genes related to cancer pathways. Collectively, our results essentially highlight the importance of and change the way in which *HPRT1*’s function is studied in biology, warranting careful examination of its role in cancer.

## 1. Introduction

Hypoxanthine phosphoribosyl transferase 1 (*HPRT1*) codes for the HPRT enzyme, a protein essential in providing the necessary building blocks for future cell growth. Through the salvage pathway, HPRT recycles nucleotides that are vital for the growth and synthesis of cells, explaining the abundant presence of HPRT in most tissues [1]. More specifically, HPRT catalyzes the conversion of hypoxanthine to inosine monophosphate, and guanine to guanosine monophosphate by transferring the 5-phosphoribosyl group from 5-phosphoribosyl 1-pyrophosphate [2]. Although widely considered to be a housekeeping gene, recent studies have shown *HPRT1* expression levels to be highly variable between malignant and normal tissue, suggesting that it is unreliable as an endogenous control in cancer-related studies [3], and also indicating its plausible role in cancer biology. *HPRT1* expression levels varied widely in a large cohort of both normal and malignant samples of different tissue origin, with breast cancer exhibiting the highest average *HPRT1* compared to other malignancies [4]. Studies from various cancers, such as colorectal cancer, have shown elevated levels of *HPRT1* in tumor samples compared to normal tissues [3,4,5]. Additionally, the same study found *HPRT1* localizes to the surface of the cell membrane in more progressive types of colorectal cancer [4,5]. Likewise, prostate cancer patient samples showed an increased expression of *HPRT1* when compared to normal tissues [3]. These observations prompted this investigation of *HPRT1* as a potential biomarker, including its role in breast cancer.

Interestingly, we also found *HPRT1* expression to be highest in the basal subtype of breast cancer patients, which is the most aggressive cancer and remains a difficult molecular subtype to treat due to its heterogeneity [6]. Among available therapies, the non-targeted nature of treatment options, especially in endocrine receptor-negative cancers, can result in devastating health consequences, side effects, and a notable risk of relapse [7]. With the knowledge that HPRT protein levels are significantly upregulated in basal subtype breast cancer compared to normal samples, we performed gene expression analysis and identified *HPRT1*-regulated pathways that are possibly crucial in breast cancer biology. Here, we also evaluated its diagnostic potential in breast and other cancer types. Our results revealed that *HPRT1* expression is elevated in a majority of the cancers, including breast cancer, and is a critical regulator of cellular pathways associated with rapid cell growth in basal subtype breast tumors. Additionally, higher *HPRT1* expression predicts poorer overall survival in a variety of cancer patients, which signifies its clinical value. Overall, these results reveal essential aspects of *HPRT1*’s role, which was thought to be a housekeeping gene.

## 2. Results

### 2.1. HPRT1 Is Highly Expressed in All Cancer Types

**Figure 1:**
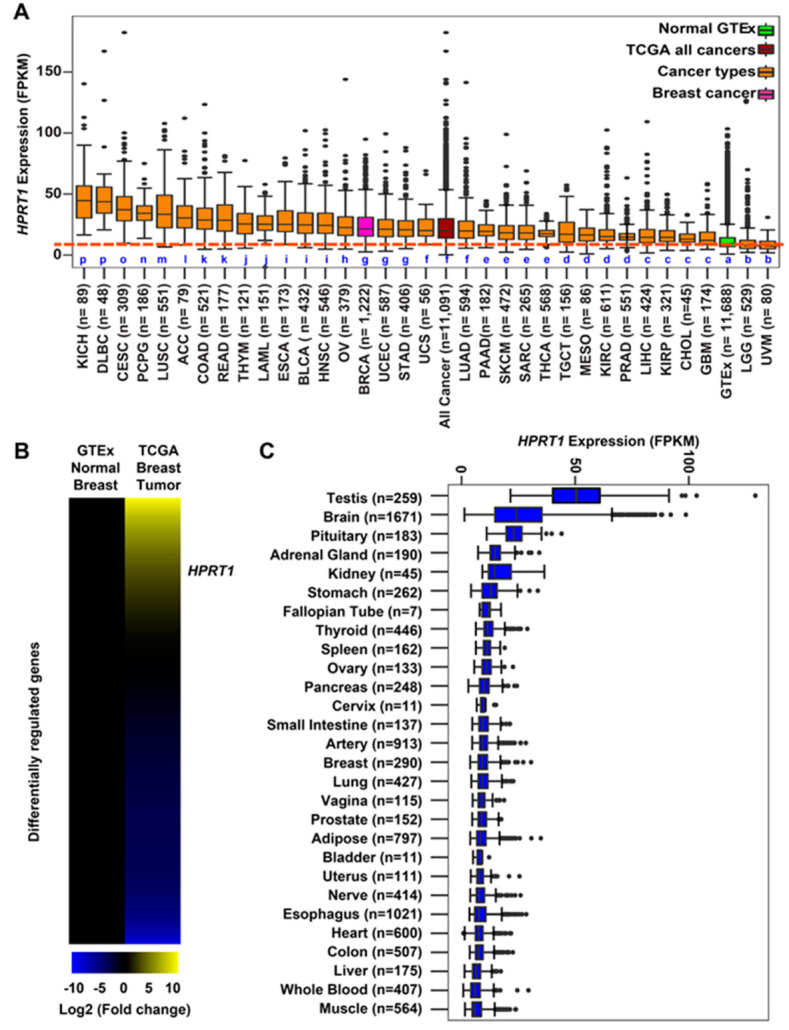
Hypoxanthine phosphoribosyl transferase 1 (*HPRT1*) expression across multiple cancer types. (**A**) Expression of *HPRT1* (fragments per kilobase of million mapped reads (FPKM)) comparing all cancer types from The Cancer Genome Atlas (TCGA) (Table S1), aggregate TCGA samples, and normal samples from Genotype-Tissue Expression (GTEx). Box plots marked with different letters in blue (a, b, c, d, e, f, g, h, i, j, k, l, m, n, o, and p) are significantly different from each other (Wilcoxon rank-sum test). (**B**) Heatmap depicting differential expression of genes (*HPRT1*–labeled) comparing normal breast tissue from GTEx against breast tumor samples from TCGA. The scale goes from bright blue to bright yellow corresponding to the log_2_ fold change (−10 to 10, respectively). (**C**) Expression of *HPRT1* (FPKM) in normal tissue samples from GTEx.

**Figure 2:**
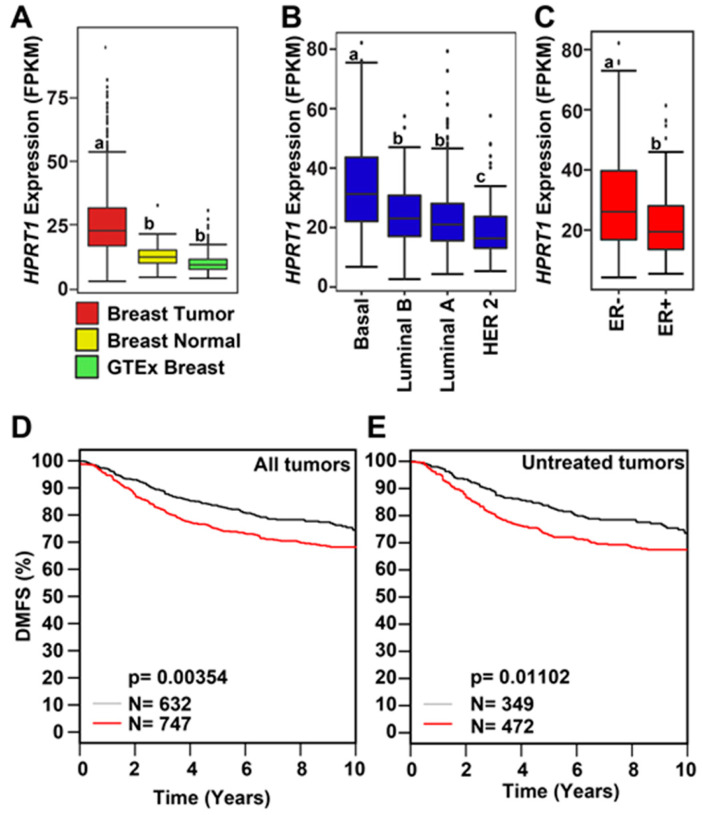
*HPRT1* expression in breast cancer. (**A**) Expression of *HPRT1* (FPKM—Fragments per Kilobase of transcript per Million mapped reads) comparing data from The Cancer Genome Atlas (TCGA) against normal breast samples from TCGA and Genotype-Tissue Expression (GTEx), respectively. Box plots marked with different letters (a, b) are significantly different from each other (*p* < 2.22 × 10^−16^; Wilcoxon rank-sum test). (**B**) Expression of *HPRT1* (FPKM) comparing different molecular subtypes of breast cancer: basal, luminal B, luminal A, and HER2. Box plots marked with different letters (a, b, c) are significantly different from each other (*p* < 2.3 × 10^−7^ and *p* < 0.041; Wilcoxon rank-sum test). (**C**) *HPRT1* expression (FPKM) grouped by estrogen receptor (ER) + and (ER)− subtypes. Box plots marked with different letters (a, b) are significantly different from each other (*p* < 0.00018; Wilcoxon rank-sum test). Kaplan–Meier curve showing survival analysis and poor clinical outcome in all (**D**) and untreated (**E**) breast cancer patients in terms of DMFS (distant metastasis-free survival; red line—higher expression; black line—lower expression). The cancer outcome linked gene expression data were accessed and graphed in March 2020, using http://co.bmc.lu.se/gobo/.

The expression of *HPRT1* was computed in fragments per kilobase of million mapped reads (FPKM) for individual cancer types as well as aggregated across all cancer types from The Cancer Genome Atlas (TCGA). *HPRT1* expression in FPKM was also aggregated across all normal tissues from the Genotype-Tissue Expression (GTEx) consortium (Figure 1A). The expression of *HPRT1* was elevated when aggregated across cancer types in comparison to its expression aggregated across normal tissues (Figure 1A). Likewise, *HPRT1* expression levels were elevated in individual cancer types derived from different organs compared to its expression aggregated across normal tissues, showing significant elevation in cancers, including those of the kidneys, lung, colon, esophagus, bladder, and breast, amongst others (Figure 1A). Notably, *HPRT1* expression was upregulated in breast cancer samples, prompting interest in further examining the role of *HPRT1* in this cancer (Figure 1B). Of note, *HPRT1* was found to be highly expressed in testis when compared to other normal tissue types in a manner similar to many cancer/testis genes (CT genes) (Figure 1C). A comparison of breast tumor and normal breast tissue showed significantly higher *HPRT1* expression in breast tumor samples when compared to normal TCGA and GTEx breast (Figure 2A). Analysis of *HPRT1* expression in different subtypes of breast cancer showed that the basal subtype had the highest *HPRT1* expression, followed by luminal B, luminal A, and HER2 (human epidermal growth factor receptor 2), respectively (Figure 2B), with levels being highest in estrogen receptor negative (ER-) tumors compared to estrogen receptor positive (ER+) tumors (Figure 2C). On the basis of these findings, we hypothesized that *HPRT1* expression predicts patient outcomes, and we used the Kaplan–Meier estimator to determine disease progression through distant metastasis-free survival (DMFS). The results showed that over time, breast tumors with higher *HPRT1* expression had shorter DMFS, corresponding to poorer clinical outcomes (Figure 2D). Additionally, among patients with untreated tumors, those with higher *HPRT1* expression exhibited shorter DMFS (Figure 2E). Altogether, these results suggest that *HPRT1* is associated with breast cancer progression and is a potential prognostic marker.

### 2.2. HPRT1 Regulates Cancer-Related Pathways in Triple-Negative Breast Cancer

**Figure 3:**
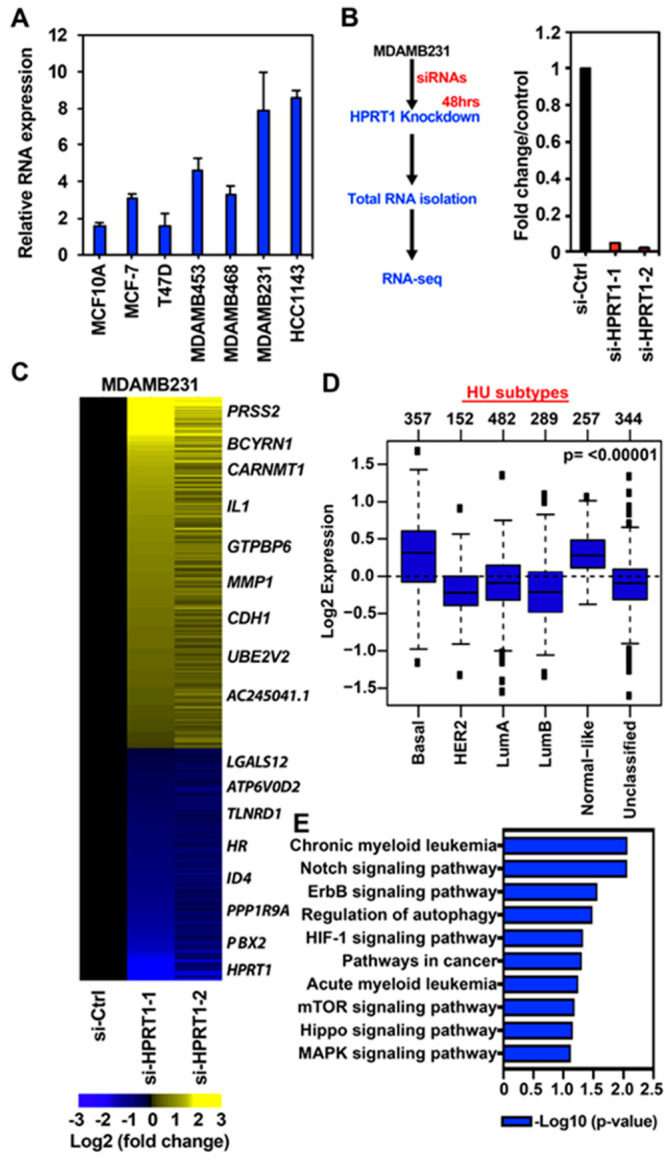
*HPRT1* regulates cancer-related pathways. (**A**) Expression of *HPRT1* in different breast cancer cell lines. (**B**) Knockdown of *HPRT1* using two different siRNAs in the triple-negative MDAMB231 breast cancer cell line. (**C**) Heatmap showing differential expression of genes upon HPRT1 knockdown. (**D**) The aggregate expression of *HPRT1* and top *HPRT1*-downregulated genes (≤−2 fold) preferentially expressed in basal breast tumor samples. Observed differences are significant, as determined by an ANOVA comparison of the means (*p*-value < 0.00001); the cancer outcome-linked gene expression data were accessed and graphed on March 2020 using the online tool http://co.bmc.lu.se/gobo/. (**E**) Gene Ontology (KEGG pathways) categories showing the highest enrichment in a list of downregulated genes obtained after RNA-seq analysis of *HPRT1* knockdown in MDAMB231 cells. GO (gene ontology) terms are indicated on the *y*-axis. *p*-value at *x*-axis indicates the significance level of each pathway, as obtained from the online DAVID tool.

To identify *HPRT1*-regulated pathways in basal breast cancer, we evaluated the relative RNA expression of *HPRT1* in cell lines representing different molecular subtypes of breast cancer. The non-tumorigenic cell line MCF10A had the lowest *HPRT1* expression, and it increased across the ER+/luminal A cell lines MCF-7 and T47D, and MDAMB453 (ER-, luminal) to the basal subtypes/triple-negative MDAMB468 and MDAMB231, and HCC1143 (Figure 3A). Since *HPRT1* expression was highest in the basal molecular subtype of breast cancer, we chose the MDAMB231 breast cancer cell line as a representative to understand *HPRT1*’s role in regulating key tumorigenic genes and pathways. The knockdown of *HPRT1* was performed using two different siRNAs in the triple-negative MDAMB231 breast cancer cell line (Figure 3B and Figure S1A), and we performed differential gene expression analysis of RNA-seq data (Figure 3B). The results showed that knockdown of *HPRT1* in the MDAMB231 cell line affected a significant number of genes; knockdown with si-HPRT1-1 altered 7408 genes, si-HPRT1-2 altered 4409 genes, and both siRNAs co-altered the expression of 2787 genes (Figure S2A, top panel). Co-regulated genes were further categorized into upregulated and downregulated (Figure S1B, MDAMB231). Volcano plots revealed si-HPRT1-1 up/down-regulated an almost equal number of genes, while si-HPRT1-2 treatment led to more genes becoming upregulated than downregulated (Figure S2B, top panel). The genes that were differentially regulated by both siRNAs were chosen, and a heatmap was generated to show the prominently regulated genes (Figure 3C). These genes were selected on the basis of log2 fold change compared to the control siRNA, including genes such as *PRSS2* (serine protease 2) and *CDH1* (Figure 3C).

**Figure 4:**
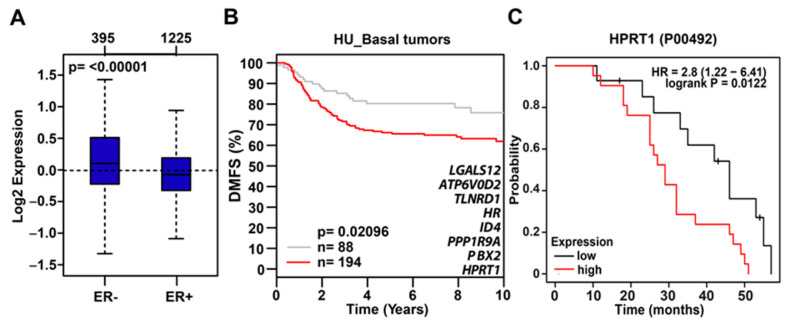
*HPRT1*-downregulated gene expression profile in ER- tumors. (**A**) The aggregate expression of *HPRT1* and top *HPRT1*-downregulated genes (≤−2 fold) preferentially expressed in ER- breast tumor samples. Observed differences are significant as determined by an ANOVA comparison of the means (*p*-value < 0.00001). (**B**) Kaplan–Meier analysis (DMFS) using the aggregate expression of *HPRT1* and top *HPRT1*-downregulated genes (≤−2 fold) showed poor clinical outcome in basal breast cancer patients (red line—higher expression or grey line—lower expression). The cancer outcome linked gene expression data was accessed and graphed in March 2020 using the online tool http://co.bmc.lu.se/gobo/. (**C**) Kaplan–Meier analysis (overall survival) of HPRT1 protein abundance showed poor clinical outcome in triple-negative breast tumors (patient data past 60 months were excluded from the analysis). The cancer outcome-linked protein expression data were accessed and graphed on 4 June 2020, using www.kmplot.com.

Interestingly, tumor suppressor gene *CDH1* was upregulated upon *HPRT1* knockdown, reiterating *HPRT1*’s role in cancer biology. The top seven *HPRT1*-downregulated genes shown in the heatmap were evaluated across different molecular subtypes and were found to be preferentially expressed in basal breast cancer molecular subtype tumor samples (Figure 3D). In addition, gene ontology (KEGG pathways) categories were enriched for pathways related to cancer, CML, Notch, ErbB, mTOR, Hippo, and MAPK signaling (Figure 3E). A majority of the significantly differentially regulated genes (based on *p*-value) interestingly belong to the Notch and ErbB signaling pathways (Figure 3E). Among genes downregulated by *HPRT1* knockdown in MDAMB231, their expression was higher in ER- than ER+ breast tumors. This finding correlates with data showing that these genes (positively regulated by *HPRT1*) are more highly expressed in basal type ER- patient tumor samples, and when compared to ER+ tumor types (Figure 4A) have lower DMFS, and poorer clinical outcome (Figure 4B). Further, overall survival analysis based on HPRT1 protein expression in triple-negative breast cancer patients suggested its association with clinical outcome (Figure 4C).

### 2.3. HPRT1 Knockdown in Normal Breast and ER+ Breast Cancer Cell Line

In addition to basal cell type, we investigated the effect of *HPRT1* knockdown in MCF10A (normal breast) and MCF-7 (ER+ breast cancer) cells (Figure S1C,D) using global transcriptomic profiling (Figure S1C). Intriguingly, the impact of *HPRT1* on gene regulation was not as dramatic in MCF10A and MCF-7 cells when compared to MDAMB231 cells, as evident in the Venn diagram, volcano plot, and heatmap of differentially expressed genes (Figure 3, Figures S2 and S3). There were a mere 158 (MCF10A) and 41 (MCF-7) differentially regulated genes affected by both siRNAs, and interestingly, a large portion of the differentially regulated genes were upregulated (Figures S2 and S3).

Additional box plot representation showed the fold change in downregulated genes when compared to upregulated genes (Figure S1E), and these results agree with the above data showing not many genes being affected, especially in the MCF-7 cells (Figure S1E). These molecular analyses closely correlated with the effect of *HPRT1* expression on clinical outcome, which confirmed the subtype-specific role of *HPRT1* in breast cancer.

**Figure 5:**
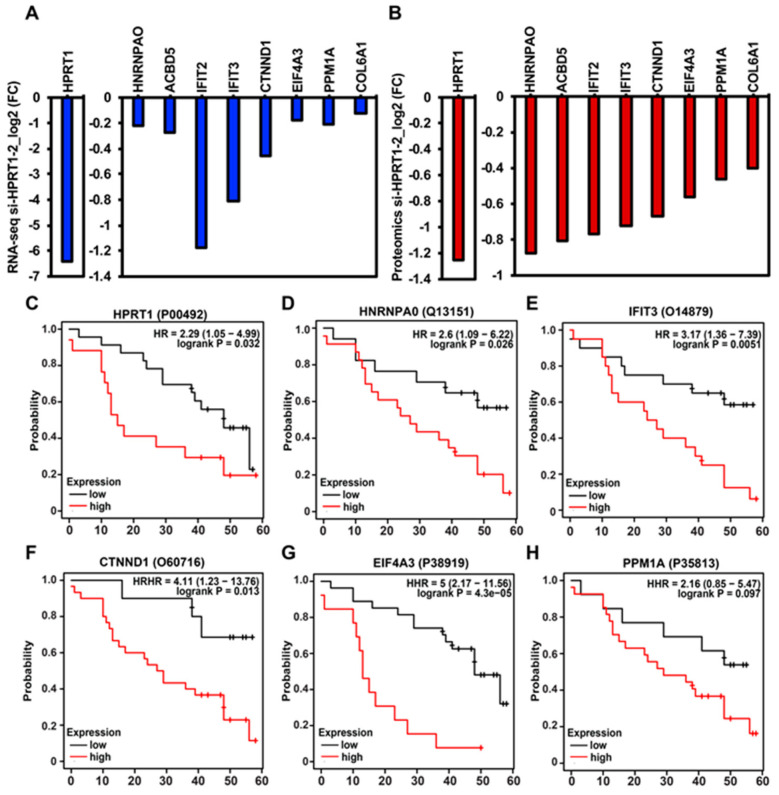
Tandem mass tag-mediated quantification of *HPRT1*-downregulated proteins, and their clinical value. (**A**) Expression of *HPRT1*-downregulated genes, as assessed by RNA-seq upon *HPRT1* knockdown using si-HPRT1-2 in MDAMB231 cells that overlapped with the *HPRT1*-downregulated proteins identified by mass spectrometric analysis. (**B**) Expression of the top 10 overlapping (RNA-seq and mass-spec, combined analysis) downregulated genes upon *HPRT1* knockdown using si-HPRT1-2 in MDAMB231 cells. (**C**) Kaplan–Meier analysis (overall survival) of HPRT1 protein abundance showed poor clinical outcome in breast cancer patients. (**C**–**H**) Kaplan–Meier analysis (overall survival) of HPRT1-downregulated proteins shows poor clinical outcome in breast cancer patients (patient data past 60 months were excluded from the analysis). The cancer outcome-linked protein expression data were accessed and graphed on 4 June 2020, using (www.kmplot.com).

Further, to integrate the *HPRT1*-dependent transcriptome with the *HPRT1*-regulated proteome, we performed mass spectrometric analysis using a powerful TMT (tandem mass tag) technique upon *HPRT1* knockdown using a siRNA (si_HPRT1-2) in MDAMB231 cells. Identified peptides were quantified, and protein abundance was determined by proteome discover 2.2. Similarly, as we saw in the decrease of *HPRT1* RNA as assessed by RNA-seq (Figure 5A), a decrease in HPRT1 protein was observed in the mass spectrometric analysis (Figure 5B). For subsequent analysis, we used proteins that were downregulated <−0.4-fold at the protein expression level, and also downregulated in RNA-seq analysis (HPRT1, Heterogeneous Nuclear Ribonucleoprotein A0-HNRNPA0, ATP Binding Cassette Subfamily G Member 5-ACBD5, Interferon Induced Protein With Tetratricopeptide Repeats 2-IFIT2, Interferon Induced Protein With Tetratricopeptide Repeats 3-IFIT3, Catenin Delta 1-CTNND1, Eukaryotic Translation Initiation Factor 4A3-EIF4A3, Protein Phosphatase Mg2+/Mn2+ Dependent 1A-PPM1A; Table S2). Interestingly, protein abundance of HPRT1, HNRNPA0, IFIT3, CTNND1 and EIF4A3 predicted poor overall survival in breast cancer patients, however, PPM1A association was non-significant (Figure 5C–H). Collectively, these results clearly showed a causal relationship between HPRT1 expression and basal breast cancer.

### 2.4. Clinical Value of HPRT1 in Other Cancer Types

**Figure 6:**
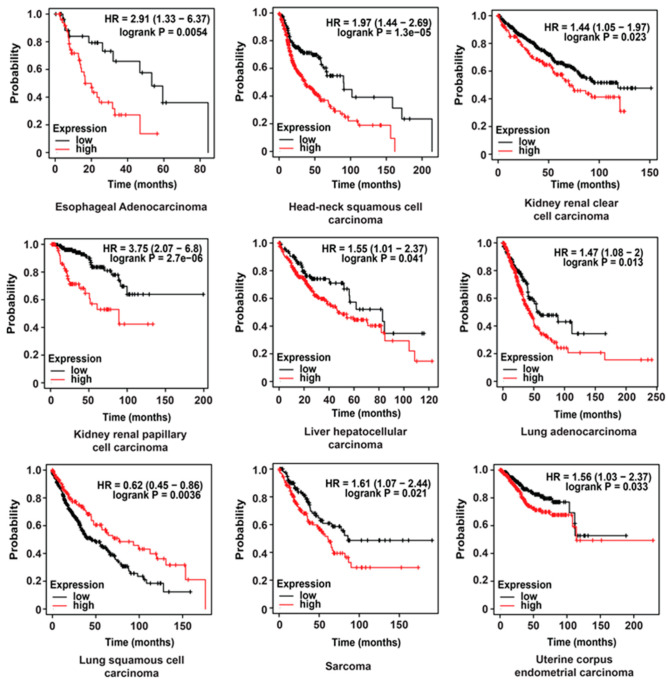
Clinical value of *HPRT1* expression in cancers. Kaplan–Meier overall survival analyses of patients expressing higher (red line) or lower (black line) levels of *HPRT1* RNA evaluated across pan-cancer types. The cancer outcome-linked gene expression data were accessed and graphed on 2 June 2020, using www.kmplot.com.

The prognostic value of *HPRT1* in other cancer types was evaluated using a pan-cancer database [8]. We generated Kaplan–Meier plots using samples of esophageal adenocarcinoma, head-neck squamous cell carcinoma, kidney renal clear cell carcinoma, kidney renal papillary cell carcinoma, liver hepatocellular carcinoma, lung adenocarcinoma, lung squamous cell carcinoma, sarcoma, and uterine corpus endometrial carcinoma patient samples. We observed elevated levels of *HPRT1* RNA to be indicative of poor overall survival in all the cancers above except for lung squamous cell carcinoma (Figure 6). Altogether, these results suggest that *HPRT1* is a biomarker with prognostic value in a majority of cancer types.

## 3. Discussion

*HPRT1* is considered a housekeeping gene and is widely used as an endogenous control in gene expression studies; however, recent studies, including our data, have revealed its potential involvement in cancer [3,4,5]. Unchecked and aberrant exponential cell growth, a hallmark of cancer, results in increased transcriptional output related to cancer genes. To meet the increased demand, *HPRT1,* a housekeeping gene under normal cellular conditions, could now plausibly be required at higher levels; hence, *HPRT1* could be differentially expressed in cancers.

Our analysis clearly demonstrated that *HPRT1* expression was significantly upregulated in breast cancer, most notably in the basal subtype (Figure 2). Breast tumors with higher *HPRT1* expression showed a worse clinical outcome, and its knockdown in the triple-negative basal-type MDAMB231 cells showed significant alteration in the expression of genes involved in Notch and ErbB signaling pathways (Figure 2 and Figure 3). Further, *HPRT1*’s role in nucleotide recycling, which is critical in providing the building blocks for the uncontrolled proliferation of cancer cells, indicates its function in cancer [9]. Upon further evaluation, we also found *HPRT1* expression to be higher in other carcinomas and adenocarcinomas and observed that higher *HPRT1* expression is closely associated with poorer clinical outcomes (Figure 6). There were, however, some exceptions in brain lower grade glioma (LGG) and uveal melanoma (UVM) (Figure 1). *HPRT1*’s increased expression level across several cancer types suggest that *HPRT1* could act as a potential prognostic marker [3].

## 4. Materials and Methods

### 4.1. Cell Culture

Estrogen receptor-positive human breast cancer cell line MCF-7 [10] was maintained in Eagle’s essential medium (Sigma-Aldrich, Saint Louis, MO, USA, M1018) and was supplemented with 5% calf serum (Sigma-Aldrich, N4637; 10,000 units/mL of penicillin, 10,000 μg/mL streptomycin, 1 M HEPES-hydroxyethyl piperazineethanesulfonic acid, and gentamycin at 10 mg/mL). MCF10A cell line (ATCC CRL-10317), a non-tumorigenic epithelial cell line, was grown in a serum-free mammary epithelial cell growth medium (VWR, C-21010B) supplemented with Supplement Mix and 100 ng/mL cholera toxin (Sigma-Aldrich, C8052). MDAMB453 (ATCC HTB-131) luminal cells, which are androgen receptor-positive, but negative for estrogen receptor-α, progesterone receptor, and HER-2, were maintained using Dulbecco’s modified medium (high glucose) (Sigma-Aldrich, D6429) with 10% fetal bovine serum (Fisher Scientific, Hampton, NH, USA, 26140079). The other TNBC (triple-negative breast cancer) cell lines MDAMB468 (ATCC HTB-132) and HCC1143 (ATCC CRL2321) were maintained on Roswell Park Memorial Institute (RPMI) 1640 medium (Sigma-Aldrich, R8758) and 10% fetal bovine serum (Fisher Scientific, 26140079). MDAMB231 cells (ATCC HTB-26) were maintained in Dulbecco’s modified medium (high glucose) (Sigma-Aldrich, D6429) with 10% fetal bovine serum (Fisher Scientific, 26140079; 10,000 units/mL of penicillin and 10,000 μg/mL streptomycin). All the cell lines were maintained with 5% CO_2_ at 37 °C.

### 4.2. HPRT1 Silencing Experiments

MCF10A, MCF-7, and MDAMB231 cells were seeded to ~60% confluency, and human *HPRT1* siRNAs from Dharmacon (Cat. ID: MQ-008735-02-0002) and control siRNA from Sigma (Cat. No. SIC001 MISSION siRNA Universal Control) were used for knockdown experiments. si-HPRT1-1 (GUUUAUUCCUCAUGGACUA), si-HPRT1-2 (GACUGAACGUCUUGCUCGA), and si-Ctrl were transfected at 10 nM concentration with RNAiMax Lipofectamine reagent, following the manufacturer’s recommendations. Experiments were performed in biological quadruplets. Total RNA was extracted from cells 48 h post siRNA transfection, using the EZ-10 DNAaway RNA Miniprep kit (Bio Basic, BS88136) according to the manufacturer’s instructions. RNA concentration was measured via nanodrop, and cDNA was synthesized using M-MLV Reverse Transcriptase (Promega, M170B) according to the manufacturer’s protocol. Relative expression was evaluated with quantitative real-time PCR using PowerUP SYBR Green Master Mix (Thermo Fisher Scientific, Waltham, MA, USA, A25743) in Applied Biosystems StepOnePlus Real-Time PCR system (Thermo Fisher Scientific).

Primers for RT-qPCR

*HPRT1* forward primer: 5′-AGAAGTTTTGTTCTGTCCTGGAA-3′, 

*HPRT1* reverse primer: 5′-GGGAACTGCTGACAAAGATTCAC-3’

*RPL19* forward primer: 5′-ACATCCACAAGCTGAAGGCA-3′ 

*RPL19* reverse primer: 5′-TGCGTGCTTCCTTGGTCTTA-3′ 

### 4.3. Total RNA Isolation, RNA-Seq Library Preparation, and Sequencing

For RNA-seq analyses, two biological replicates were sequenced for each of the conditions (si-Ctrl, si-HPRT1-1, and si-HPRT1-2) in MCF10A, MCF-7, and MDAMB231 cells. Total RNA was isolated according to the manufacturer’s instructions (EZ-10 DNAaway RNA Miniprep kit Bio Basic, BS88136). RNA was eluted with RNAse-free water and assayed for quality by electrophoresis (Agilent RNA ScreenTape). Only samples with RIN (RNA integrity number) values of >9 were included in subsequent analysis. RNA-seq libraries were prepared and sequenced at Novogene Corporation.

### 4.4. Quality Control, Assembly of Transcriptome Data, and Differential Gene Expression

Adapters were trimmed from raw reads at the de-multiplexing step. Further quality control was performed using the Trim Galore package to remove low-quality reads (Phred score < 25). Only reads with intact mate pairs were retained for further analysis. Paired-end reads were then mapped to the human genome (hg38) using the HISAT2 aligner. Read counts were generated from alignments using the Feature Counts package with gencode (v28) as the reference annotation. Normalization and differential gene expression analyses were performed using the DESEQ2 package in R. Differentially expressed genes were extracted by applying *p*-value (<0.05) and base mean (>20) cutoffs. Only genes that were differentially regulated by both siRNAs were considered for further analysis. Post-sequencing analyses were performed by Kinsight Bio Analytics LLC.

### 4.5. Expression of HPRT1 in GTEx Samples

Samples from the Genotype-Tissue Expression (GTEx) database version 7 were accessed through the GTEx website (https://www.gtexportal.org) with a total of 11,688 RNA-seq available samples. TPMs (transcripts per million) and FPKMs (fragments per kilobase of transcript per million mapped reads) were calculated from gene TPMs and gene read counts, respectively. Box plots were generated using R version 3.5.2 and the R package ggplot2 using the functions ggplot, stat_boxplot, and geom_boxplot. All data were processed locally using custom Perl, R, and Bash scripts. The *HPRT1* expression (Figure 1C) data used for the analyses described in this manuscript were obtained from the GTEx Portal on 04/11/2020 or dbGaP accession number phs000424.v8.p2 on 04/11/2020 [11].

### 4.6. Expression of HPRT1 in TCGA Samples

Samples for The Cancer Genome Atlas were accessed through the Genomic Data Commons (GDC) Data Portal (https://portal.gdc.cancer.gov) [12]. Data from 33 cancer types and subtypes were accessed with a total of 11,091 samples using version 16. Datasets available with FPKM values were used for subsequent analysis. Box plots were generated using R version 3.5.2 and the R package ggplot2 using the functions ggplot, stat_boxplot, and geom_boxplot. All data were processed locally using custom Perl, R, and Bash scripts.

### 4.7. Gene Ontology Analyses

Transcripts with log2 fold change cutoff of 1.5 and *p*-value ≤ 0.05 were considered as significantly differentially expressed. Gene ontologies and pathways that harbored significantly expressed transcripts were identified using the DAVID Functional Annotation Tool [13,14].

### 4.8. Kaplan–Meier Analysis

To evaluate the prognostic value of *HPRT1*, we explored its expression in samples of esophageal adenocarcinoma, head-neck squamous cell carcinoma, kidney renal clear cell carcinoma, kidney renal papillary cell carcinoma, liver hepatocellular carcinoma, lung adenocarcinoma, lung squamous cell carcinoma, sarcoma, and uterine corpus endometrial carcinoma. The Kaplan–Meier plotter tool was used to plot overall survival for *HPRT1* (http://kmplot.com/analysis/index.php?p=service&cancer=pancancer_rnaseq) [8].

Kaplan–Meier plots (DMFS) were generated using the Gene Expression-Based Outcome for Breast Cancer Online (GOBO) tool (http://co.bmc.lu.se/gobo/). Gene expression levels in patient tumor samples were also assessed using the GOBO tool [15].

To evaluate the prognostic value of HPRT1 protein expression in triple-negative breast cancer patients, we used Liu et al. (2014) proteomics data from www.kmplot.com [16]. We also explored the clinical value of HPRT1, HNRNPA0, IFIT3, CTNND1, EIF4A3, and PPM1A proteins in breast cancer patients using Tang et al.’s (2018) data from www.kmplot.com [17].

### 4.9. Tandem Mass Tag (TMT)

We used the TMT approach to detect proteins regulated upon *HPRT1* knockdown using si-HPRT1-2 in MDAMB231 cells; replicate that showed HPRT1 depletion was selected for further analysis (*n* = 1). TMT is a powerful approach to identify differentially regulated proteins between different conditions. This is performed by labeling samples after digestion, and running the TMT samples on the Orbitrap Fusion Lumos mass-spectrometry platform, using an appropriate LC–MS/MS method at University of Texas Southwestern Medical Center. The proteins were identified, and peptides were quantified using Proteome Discoverer 2.2 (Table S3; TMT quantification of differentially regulated proteins upon HPRT1 knockdown). The abundance of proteins was normalized with RPL19 protein expression.

### 4.10. Accession Numbers

The accession number for the RNA-seq datasets generated for this study is NCBI GEO: GSE149768.

## 5. Conclusions

Altogether, the study suggests that gene expression data should be analyzed to reflect actual transcript levels, and should not be influenced by cellular origin or other biological factors. We found that *HPRT1* that was traditionally thought to be a housekeeping gene may actually have a role in cancer biology. It is differentially expressed across several cancers (Figure 1) and could be a potential biomarker in cancers (Figure 6). Wang et al. [18] found that the use of a single gene to normalize gene expression data will not reveal actual differences due to the variation observed throughout; instead, they encouraged the use of multiple housekeeping genes that can be tailored and normalized to specific datasets. They suggest that although no single normalization method could be used for all datasets if using housekeeping genes, a comprehensive set of housekeeping genes should be used rather than a single gene [18].

Collectively, our study suggests that *HPRT1* should be treated as a biomarker rather than as a housekeeping gene, and its differential expression during cancer could be exploited as a potential diagnostic or therapeutic target.

## Supplementary Materials

The following are available online at https://www.mdpi.com/2072-6694/12/6/1522/s1, Figure S1: *HPRT1* knockdown in normal breast and breast cancer cells. *HPRT1* expression upon knockdown as assessed by RNA-seq in MDAMB231 (A) and MCF10A or MCF-7 cells (D). Box plot showing the effect on cumulative gene regulation upon *HPRT1* depletion (fold change/control) in MDAMB231 (B), MCF10A or MCF-7 cells (E). (C). Knockdown of *HPRT1* using two different siRNAs in MCF10A (normal breast) and MCF-7 (ER+ breast cancer) cells, Figure S2: *HPRT1*-regulated genes in MDAMB231, MCF10A and MCF-7 cells, Figure S3: *HPRT1* knockdown in normal breast and ER+ breast cancer cell line. Heat map showing differential expression of genes upon *HPRT1* knockdown in MCF10A and MCF-7 cells, Table S1: TCGA cancer types, Table S2: Overlapping set of downregulated genes at RNA and protein level upon *HPRT1* knock down, Table S3: TMT quantification of differentially regulated proteins upon *HPRT1* knock down.
