# Supplementary material for: Hypoxanthine Phosphoribosyl Transferase 1 Is Upregulated, Predicts Clinical Outcome and Controls Gene Expression in Breast Cancer
Source: Cancers (Basel). 2020 Jun 10;12(6):1522. doi: 10.3390/cancers12061522 (PMC7352670; doi:10.3390/cancers12061522)
Supplement: Supplementary file 1 [file cancers-12-01522-s001.zip › cancers-813885-suppl-1.pdf]

Supplementary Materials: Hypoxanthine Phosphoribosyl Transferase 1 is Upregulated, Predicts Clinical Outcome and Controls Gene Expression in Breast Cancer

Melina J. Sedano, Enrique I. Ramos, Ramesh Choudhari, Alana L. Harrison, Ramadevi Subramani, Rajkumar Lakshmanaswamy, Mina Zilaie and Shrikanth S. Gadad

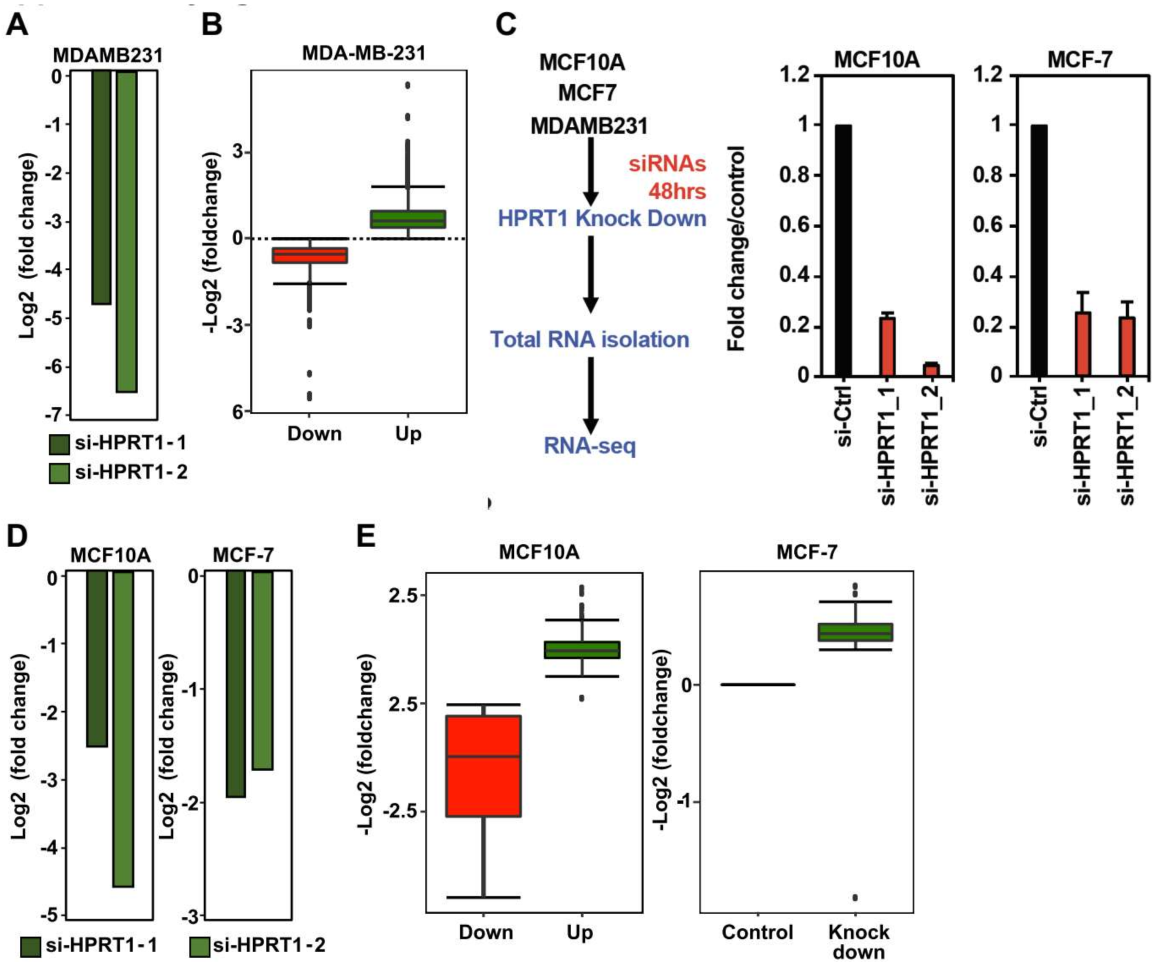

Figure S1. *HPRT1* knockdown in normal breast and breast cancer cells. *HPRT1* expression upon knockdown as assessed by RNA-seq in MDAMB231 (**A**) and MCF10A or MCF-7 cells (**D**). Box plot showing the effect on cumulative gene regulation upon *HPRT1* depletion (fold change/control) in MDAMB231 (**B**), MCF10A or MCF-7 cells (**E**). **C**. Knockdown of *HPRT1* using two different siRNAs in MCF10A (normal breast) and MCF-7 (ER+ breast cancer) cells.

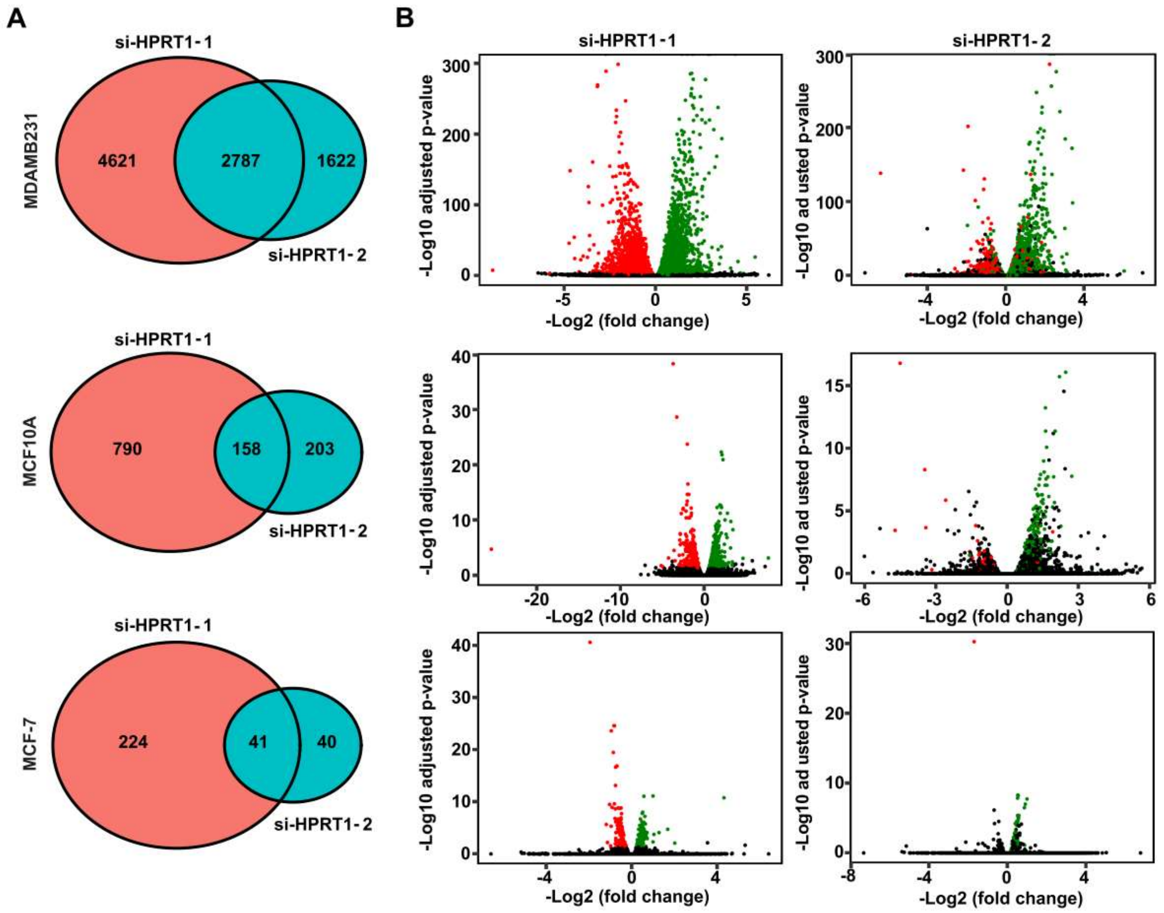

Figure S2. *HPRT1*-regulated genes in MDAMB231, MCF10A and MCF-7 cells. (**A**). Venn diagram showing the overlap of regulated genes upon *HPRT1* knockdown using two different siRNAs in MDAMB231, MCF10A, and MCF-7 cells. (**B**). Volcano plot showing the extent of individual gene regulation upon *HPRT1* knockdown based on p-value in MDAMB231, MCF10A, and MCF-7 cells.

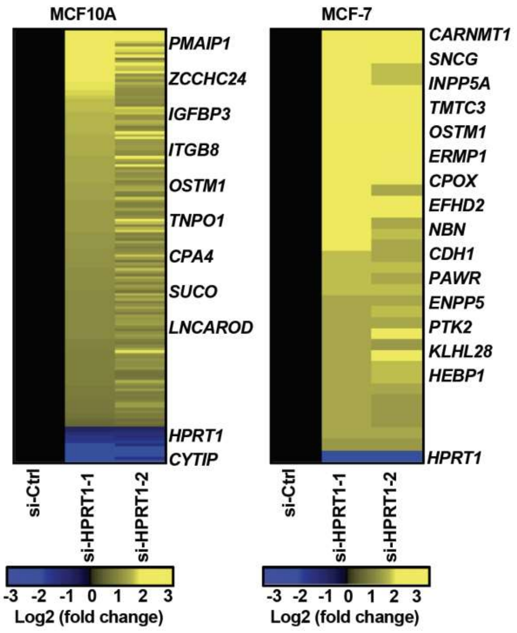

Figure S3. *HPRT1* knockdown in normal breast and ER+ breast cancer cell line. Heat map showing differential expression of genes upon *HPRT1* knockdown in MCF10A and MCF-7 cells.

Table S1. TCGA cancer types.

Abbreviation	Cancers
LAML	Acute Myeloid Leukemia
ACC	Adrenocortical carcinoma
BLCA	Bladder Urothelial Carcinoma
LGG	Brain Lower Grade Glioma
BRCA	Breast invasive carcinoma
CESC	Cervical squamous cell carcinoma and endocervical adenocarcinoma
CHOL	Cholangiocarcinoma
COAD	Colon adenocarcinoma
ESCA	Esophageal carcinoma
GBM	Glioblastoma multiforme
HNSC	Head and Neck squamous cell carcinoma
KICH	Kidney Chromophobe
KIRC	Kidney renal clear cell carcinoma
KIRP	Kidney renal papillary cell carcinoma
LIHC	Liver hepatocellular carcinoma
LUAD	Lung adenocarcinoma
LUSC	Lung squamous cell carcinoma
DLBC	Lymphoid Neoplasm Diffuse Large B-cell Lymphoma
MESO	Mesothelioma
OV	Ovarian serous cystadenocarcinoma
PAAD	Pancreatic adenocarcinoma
PCPG	Pheochromocytoma and Paraganglioma
PRAD	Prostate adenocarcinoma
READ	Rectum adenocarcinoma
SARC	Sarcoma
SKCM	Skin Cutaneous Melanoma
STAD	Stomach adenocarcinoma
TGCT	Testicular Germ Cell Tumors
THYM	Thymoma
THCA	Thyroid carcinoma
UCS	Uterine Carcinosarcoma
UCEC	Uterine Corpus Endometrial Carcinoma
UVM	Uveal Melanoma
GTEx	Genotype-Tissue Expression

© 2020 by the authors. Licensee MDPI, Basel, Switzerland. This article is an open access article distributed under the terms and conditions of the Creative Commons Attribution (CC BY) license (http://creativecommons.org/licenses/by/4.0/).
